# 

**Published:** 1870-02

**Authors:** 


### SPECIAL NOTICES.

---

Dental Chairs.—Among the great variety of operating chairs in the market, there is, perhaps, none possessing greater advantages than Butler's. This chair is said, by those who have used it, to have no superior; and, from the slight observation which we have been able to make, we are disposed to think the claim a valid one.

Geo. Watt & Co. have them, of every style, at manufacturer's prices. They have also Butler's operating case and spittoon—both very good and neat fixtures.

Southern Dental Depot, New Orleans, La.—We have just received the business circular of this house, and we must congratulate the profession of the South on having so good facility for obtaining Dental materials. This can not be otherwise, since Dr. Chas. E. Kells is at the head of it. The Doctor was a successful practitioner for nearly twenty years, and his ability for furnishing the profession with what they require, is inferior to none. Subscriptions for the Dental Register will be received by that house, and their receipts for subscriptions or advertising will always be valid. T.

The commencement exercises of the Ohio College of Dental Surgery, will be held in the Lecture-room of the College, on Wednesday evening, March 2d, at 7½ o'clock. The annual address will be delivered by Dr. W. H. Morgan, of Nashville, Tennessee. A cordial invitation is extended all the members of the Dental and Medical professions, and all others, to be present.

The proposition in referenee to the Engagement Book, made on our Special Notice page, in Jan. No., should have stated that that proposition extends only to the 1st of April next. We hope all who wish the Engagement Book under that proposition will not overlook this statement.

AMERICAN DENTAL ASSOCIATION—Meets on the first Tuesday in August next year at Nashville, Tenn. *Pres.* H. JUDD, St. Louis. *Rec. Sec.* M. S. DEAN, Chicago.

AMERI AN DENTAL C NVENTION—Meets in New York, Sept. 1870 *Pres.* Dr. J. G. AMBLER. *Sec.* and *Treas* Dr. J. H. SMITH, New Haven, Conn

*List of Societies represented in the Ameri an Dental Association.*

AMERICAN DENTAL CONVENTION—Meets annually. *Pres.* J. M. CROWELL of New York, *Rec.* and *Cor Sec* J. S. LATIMER, of New York.

AMERICAN ACADEMY OF DENTAL SCIENCE—Meets at Boston, Mass. *Pres.* DANIEL HARWOOD. M. D., of Boston. *Sec.* E. N HARRIS, D. D. S., of Boston.

BROOKLYN DENTAL SOCIETY—Meets semi-monthly. *Pres.* C. D. Cook. *Rec. Sec.* E L CHILDS *Cor. Sec* WM. JARVIE Jr.

BUCKS COUNTY DENTAL ASSOCIATION, (PA.) Meets semi-annually on the first Monday in May and November. Next meeting at Newton, in November. *Pres.* H. P. Yerkes, *Sec.* G W. Adams.

BUFFALO DENTAL SOCIETY—Meets monthly at Buffalo. *Pres.* B. F. Whitney, *Rec. Sec.* S A. FREEMAN.

BOSTON DENTAL INSTITUTE. Meets first Tuesday of each month. *Pres.* D. G. Williams. *Cor. Sec.* D S. Dickerman

CUMBERLAND VALLEY DENTAL SOCIETY—Meets semi-annually.* *Pres.* J. L. SUESSEROTT, of Chambersburg. *Sec.* GEO. W NEIDICH, of Carlisle, Pa.

CINCINNATI DENTAL SOCIETY—Meets semi-monthly at Cincinnati. *Pres.* Geo. WATT, *Rec. Sec.* Wm. TAFT.

CENTRAL OHIO DENTAL ASSOCIATION—Meets Annually on the second Tuesday of July. *Pres.* S P. HARRINGTON, Newark. *Sec.* G. W. DECAMP Mansfield. O.

CHICAGO DENTAL SOCIETY—Meets monthly at Chicago. *Pres.* J. H. YOUNG. *Rec and Cor. Sec.* C. R E. KOCH.

CONNECTICUT VALLEY DENTAL ASSOCIATION—Meets semi-annually, second Tuesday of May and October, *Pres.* A. A. HOWLAND, Barre, Vt. *Rec. Sec.* W. H. JONES, of Boston, Mass.

CONNECTICUT STATE DENTAL ASSOCIATION—*Pres.* SAML. MALLETT, New Haven. *Cor. Sec.* JOHN CODY, Hartford. *Rec. Sec.* Dr. POWERS, Hartford.

CHARLESTON DENTAL ASSOCIATION.*—*Prest.* I. B. PATRICK. *Sec. and Treas.* T. F. CHUPEIN.

DELAWARE DENTAL ASSOCIATION—Meets semi-annually. *Pres* E, W. HAINES, Newark. *Sec.* C. R. JEFFRIES, Wilmington.

EAST TENNESSEE DENTAL ASSOCIATION—Meets annually at Knoxville, Tenn. *Pres* J. A. CRAZIER of Knoxville *Rec. Sec.* W. F. FOWLER, Tenn.

FOREST CITY SOCIETY OF DENTAL SURGEONS. *Pres* B. Strickland, Cleveland. *Rec. Sec.*, H. L Ambler, Cleveland. *Cor. Sec.*, Chas. Buffett, Cleveland.

GEORGIA STATE DENTAL SOCIETY—Next meeting at Atlanta, July 30th, 1870. *Pres.* F. Y. CLARK; *Cor. Sec.* J. P. H. BROWN, Augusta.

HARRIS DENTAL ASSOCIATION OF LANCASTER, Pa —Next meeting at Columbia, on Thursday the [illegible]th of Nov. next. *President,* SAM'L. WELCHENS. *Sec.* WM. NICHOLS AMER

HUDSON RIVER ASSOCIATION OF DENTAL SURGEONS—Meets on the second Wednesdays of May, September and January. *Pres.* L. S. STRAW, Newburg N .Y. *Rec. Sec* T. W. DuBOIS, Poughkeepsie, N. Y.

HUDSON VALLEY DENTAL ASSOCIATION—meets monthly at Troy, N. Y. *Pres.* C. H. JENKINS, *Rec. Sec.* E. J. YOUNG, *Cor. Sec.* S. D. FRENCH.

ILLINOIS STATE DENTAL SOCIETY—Meets annually. *Pres.* M S. Dean, of Chicago. *Sec.* C. S. SMITH, of Springfield.

INDIANA STATE DENTAL SOCIETY—Meets annually at Indianapolis on the last Tuesday of June. *Pres.* J. F. Johnston, of Indianapolis. *Rec. and Cor. Sec* S. B. Brown.

IOWA STATE DENTAL SOCIETY—Meets annually. *Next meeting at Iowa City, on the second Tuesday of July. *Pres.* J HARDMAN, of Muscatine. *Rec. Sec.* A. B MASON, of Waterloo. *Cor Sec.* J. P WILSON, of Burlington.

LAKE ERIE DENTAL ASSOCIATION.—Meets Annually.* *Pres.* B.T. WHITNEY, Buffalo N. Y., *Sec.* W. E. MAGILL, Erie, Pa.

LEBANON VALLEY DENTAL ASSOCIATION—*Prest.* W. K. BRENIZER, *Sec.* S. H. GUILFORD.

MAD RIVER VALLEY DENTAL SOCIETY—Meets Semi annually on the first Tuesday of May next. Next meeting at Cincinnati, O. *Pres.* J. H. PAINE, Middletown, O. *Sec.*, S. B. TIZZARD, Troy, O.

MARYLAND ASSOCIATION OF DENTISTS—Meets the last Thursday of every month. *Pres.*, J. B. BEAN, *Sec.*, S M. FIELD.

MASSACHUSETTS DENTAL SOCIETY—Meets monthly. *Pres.*, T. H. Chandler, *Rec. Sec.* A. Brown, *Cor Sec.* E. Blake.

MICHIGAN DENTAL ASOCIATION—Meets annually second Tuesday of Oct. at Jackson. *Pres.* E. S. HOLMES, of Grand Rapids; *Rec. and Cor. Sec.* G. H. MOSHER, of Jackson.

MUSKINGUM VALLEY DENTAL ASSOCIATION—Meets on first Monday of each month at Zanesville, O. *Pres.* W. M. Herriot, of Zanesville, O., *Sec.* W. W. Chapelear, Zanesville, O.

MISSISSIPPI VALLEY DENTAL ASSOCIATION—Meets annually at Cincinnati, on the first Wednesday in March. *Pres.* W. H. MORGAN, Nashville. *Rec. Sec.* C. M. WRIGHT, Cincinnati, O. *Cor. Sec.* N. W. Williams, Xenia, O.

MISSOURI DENTAL ASSOCIATION—Meets on the first Tuesday of June, in St. Louis *Pres.* H. JUDD St. Louis, *Sec* W. H. EAMES, St. Louis.

MISSOURI VALLEY DENTAL SOCIETY.* Meets on the second Tuesday in July and January *Pres.* E. J. WOODBURY of Council Bluffs, Iowa *Sec. and Treas.* J. [illegible], Sanborn, Tabor, Iowa.

MERRIMAC VALLEY DENTAL SOCIETY—Meets semi annually first Tuesday of Oc and May.* *Pres.* I. H. KIDDER, Mass. *Rec. Sec.* A. M. DUDLEY, of Lowell. *Cor. Sec.* D. T. PORTER, Lawrence, Mass.

MEMPHIS DENTAL SOCIETY—Meets on the first Tuesday of each month. *Pres* W. T. Arrington, *Sec.* V. F. Elliott. *Treas.* C. L. Harris.

NASHVILLE DENTAL ASSOCIATION—Meets on the second Tuesday of each month *Pres.* W. H. MORGAN, *Sec* J C. ROSS, *Cor. Sec.* R RUSSELL.

NEW YORK STATE DENTAL SOCIETY—Meets at Albany on the last Tuesday of July next *Pres.* A. WESTCOTT. of Syracuse. *Sec.* L. W. ROGERS, of Utica

NEW HAVEN DENTAL SOCIETY—Meets on the first Wednesday of each month at New Haven. *Pres.* J. T. METCALF, *Rec and Cor. Sec.* C. L. SMITH, of New Haven.

NEW LONDON COUNTY DENTAL SOCIETY—Meets monthly.* *Pres.* R W. BROWNE. *Rec. Sec.* WM. ALLENDER

NORTHERN OHIO DENTAL ASSOCIATION—Meets Semi-annually, (first Tuesday of May and October) in Cleveland. *Pres.* W. P. HORTON, *Rec. Sec.* H L. AMBLER, *Cor. Sec.* CHAS BUFFETT, Cleveland.

NORTHERN IOWA DENTAL ASSOCIATION—Meets annually, on the 2d Tuesday in June, 1870. Next meeting at Waterloo. *Pres.* E. S. CLARK, Dubuque. *Rec. Sec.* J. Z. ABBOTT, Manchester. *Cor. Sec.* A. B. MASON, Waterloo.

OHIO DENTAL COLLEGE ASSOCIATION—Meets annually in March, at Cincinnati *Pres.* Geo H. Cushing, Chicago, Ill. *Rec Sec.* J TAFT, of Cincinnati.

OHIO STATE DENTAL ASSOCIATION.—Meets semi-annually, at Columbus. [Nex meeting—first Tuesday of December.] *Pres.* W P. HORTON, Cleveland, *Rec. Sec.* G. W. KEELY, Oxford, O. *Cor. Sec.* C. R. BUTLER, Cleveland, O.

ODONTOGRAPHIC SOCIETY OF PENNSYLVANIA—Meets monthly in Philadelphia Next meeting, Wednesday, Sept 1st. *Pres.* J. H. McQUILLEN. *Rec. Sec.* WM. H. HOWARD. *Cor. Sec.* T. C. STELLWAGEN.

ODONTOLOGICAL SOCIETY OF NEW YORK CITY—Meets the second Tuesday of each month * *Pres.* C E. FRANCIS *Rec Sec.* C. F. IVES.

OLD COLONY DENTAL ASSOCIATION—Meets quarterly.* *Pres.* C. G. Davis. *Rec. Sec.* L. W. PUFFER, North Bridgewater, Mass. *Cor. Sec.* N. C. FOWLER, Yarmouth, Mass.

PENNSYLVANIA ASSOCIATION OF DENTAL SURGEONS—Meets monthly in Phila. *Pres.* E. WILLIAMS. *Rec. Sec* JAMES TRUMAN *Cor. Sec.* E. T. DARBY.

PITTSBURG DENTAL ASSOCIATION—Meets first Tuesday of each month in Pittsburg. *Pres* C. L. WESTENBURG *Rec. Sec.* J. D. WHITE.

POUGHKEEPSIE DENTAL SOCIETY.—Meets monthly, *Pres.* J. H. Mann, *Sec.* H. F Clark.

ST. LOUIS DENTAL SOCIETY—Meets monthly. *Pres.* ISAAC COMSTOCK. *Sec.* R. J. POORE.

SUSQUEHANNA DENTAL ASSOCIATION—Meets semi-annually*. *Pres.* C. S. BECK M. D *Rec. Sec.* R. C. BURLEW.

SOCIETY OF DENTAL SURGEONS OF THE CITY OF NEW YORK—Meets semi-monthly in New York. *Pres.* B. W FRANKLIN. *Rec. Sec.* J. S. LATIMER. *Cor. Sec.* T. H. BURRAS.

SOUTHERN STATES DENTAL ASSOCIATION.—Meets annually. Next meeting the 2d Wednesday in April, 1870, at New Orleans. *Pres.* W. T. ARRINGTON. *Rec. Sec* Prof. ANGELL, New Orleans

STATE DENTAL SOCIETY OF PENNSYLVANIA—Meets annually Third Tuesday in June * Next meeting in Pittsburg, 1870. *Pres.* T. L Buckingham of Philadelphia. *Rec. Sec.* Geo. W. Neidich *Cor. Sec.* Samuel Welchens.

TENNESSEE DENTAL ASSOCIATION.—Meets semi-annually. *Pres.* W. T. Arrington of Memphis *Sec.* Wm M. R. Johns, of Somerville

TUSCARAWAS VALLEY DENTAL SOCIETY.—The annual meeting in Nov. 1870. *Pres.* JOHN McKINLY, Uhricsville. *Sec.* L. E. DISNEY, Coshocton.

WASHINGTON CITY DENTAL ASSOCIATION—Meets on the first Monday of each month. *Pres.* R. F. HUNT. *Sec.* J. C SMITH. *Librarian,* H. N. WADSWORTH.

WESTERN NEW YORK DENTAL ASSOCIATION—Meets semi-annually.* *Pres.* FRANK FRENCH, Rochester. *Sec* W. C. BARRETT, Warsaw.

***Place of meeting fixed by appointment**

### DISTRICT DENTAL SOCIETIES OF THE STATE OF NEW YORK.

*1ST DISTRICT DENTAL SOCIETY.—*Pres.* A. L. Northrop, New York City. *Sec.* W. C Horne, New York City.

2D DISTRICT DENTAL SOCIETY.—*Pres.* W. B. Hurd, Brooklyn. *Rec. Sec.* Wm. Jarvie Jr., Brooklyn. *Cor. Sec.* L. S. Straw, Newburg

3D DISTRICT DENTAL SOCIETY.—Meets at Albany semi-annually and annually. *Pres* H. H. Young, Troy. *Sec.* W. F. Winne, of Albany.

*4TH DISTRICT DENTAL SOCIETY.—*Pres.* F. C. Rich, Saratogo Springs. *Sec.* C. A Elmore, Fort Edwards.

*5TH DISTRICT DENTAL SOCIETY.—*Pres.* G. A. Foster, Utica *Sec.* Charles Barnes, Syracuse.

*6TH DISTRICT DENTAL SOCIETY.—*Pres.* S. H. McCall, Binghampton. *Sec.* F. B. DARBY, Elmira.

*7TH DISTRICT DENTAL SOCIETY.—*Pres.* A. G. COLEMAN, Canandagua. *Sec.* H. S. MILLER, Rochester.

TH DISTRICT DENTAL SOCIETY.—*Pres.* B. T. Whitney, Buffalo. *Rec. Sec.* W. C. Barrett, Warsaw. *Cor. Sec.* T. G. Lewis, Buffalo.

* Place and time of meeting fixed by appointment.

### EXCHANGES,

ST. LOUIS MEDICAL AND SURGICAL JOURNAL,
BOSTON MEDICAL AND SURGICAL JOURNAL,
THE PACIFIC MEDICAL AND SURGICAL JOURNAL, SAN FRANCISCO.
BUFFALO MEDICAL AND SURGICAL JOURNAL,
PHILADELPHIA MEDICAL AND SURGICAL REPORTER.
CINCINNATI LANCET AND OBSERVER,
DENTAL COSMOS, PHILADELPHIA.
L'ART DENTAIRE, PARIS, FRANCE.
BRAITHWAITE'S RETROSPECT, N. Y.
THE DENTAL TIMES, PHILADELPHIA,
LADIES' REPOSITORY, CINCINNATI.
HALL'S JOURNAL OF HEALTH, N. Y,
CHICAGO MEDICAL EXAMINER.
XENIA TORCHLIGHT.
THE DETROIT REVIEW OF MEDICINE AND PHARMACY.
MEDICAL RECORD, N Y.
NASHVILLE JOURNAL OF MEDICINE AND SURGERY,
AMERICAN ECLECTIC MEDICAL REVIEW, N. Y.
AMERICAN JOURNAL OF DENTAL SCIENCE, BALTIMORE.
THE UNITED STATES MEDICAL AND SURGICAL JOURNAL. CHICAGO,
NEW ORLEANS JOURNAL OF MEDICINE.
WESTERN JOURNAL OF MEDICINE. INDIANAPOLIS, IND.
AMERICAN AGRICULTURIST, N. Y.
DENTAL OFFICE AND LABORATORY, PHILA.
BOSTON JOURNAL OF CHEMISTRY.
CINCINNATI MEDICAL REPERTORY.
CHICAGO MEDICAL INVESTIGATOR.
JOURNAL OF APPLIED CHEMISTRY, N. Y.
DRUGGISTS' CIRCULAR AND CHEMICAL GAZETTE, NEW YORK.
DER ZAHNARZT, LEIPZIG.
DEUTSCHE VIERTELJAHRSSCHRIFT, NUREMBURG.
CANADA JOURNAL OF DENTAL SCIENCE, MONTREAL.
GUARDIAN OF HEALTH AND EDUCATION, BOSTON.
HARDWICKE'S SCIENCE GOSSIP, LONDON, ENGLAND.
PACKARD'S MONTHLY, NEW YORK.
POPULAR SCIENCE REVIEW, LONDON, ENGLAND.
MISSOURI DENTAL MONTHLY ST. LOUIS.
MEDICAL BULLETIN, BALTIMORE, MD.
ECLECTIC MEDICAL JOURNAL, CINCINNATI.
TRANSACTIONS, COLLEGE OF PHYSICIANS, PHILADELPHIA.

### The Dental Register

### OF THE WEST,

*Issued Monthly, at $3 a Year in Advance.*

DEVOTED TO THE INTERESTS OF THE DENTAL PROFESSION.

EDITED BY J. TAFT AND G. WATT.

The volume commences in January and closes in December.

The Register being issued monthly is always fresh, therefore indispensable to the practitioner who desires to keep posted up with the new inventions and improvements which are daily developed. Neither expense nor effort will be spared to give value and variety to its contents. Articles will be illustrated with well executed engravings whenever they will add to the interest or assist in explanation.

Its extensive circulation and frequency of issue makes it one of the BEST DENTAL ADVERTISING MEDIUMS in the United States.

RATES OF ADVERTISING.

One page, 1 year, $50. Half page, 1 year, $30. Quarter page, or card, 1 year $20.

All advertising bills payable in advance, or at furthest after the issue of the first number containing the advertisement. All transient advertisements to be paid for in advance.

☞ CONTRIBUTIONS SOLICITED.

Address, J. TAFT, or "DENTAL REGISTER," Cincinnati Ohio.

Business Communications to

**J. TAFT, Publisher,**

No. 117 West Fourth St.

